# Effects of Non-Local Diffusion on Structural MRI Preprocessing and Default Network Mapping: Statistical Comparisons with Isotropic/Anisotropic Diffusion

**DOI:** 10.1371/journal.pone.0026703

**Published:** 2011-10-31

**Authors:** Xi-Nian Zuo, Xiu-Xia Xing

**Affiliations:** 1 Laboratory for Functional Connectome and Development, Key Laboratory of Behavioral Science, Institute of Psychology, Chinese Academy of Sciences, Beijing, China; 2 Magnetic Resonance Imaging Center, Institute of Psychology, Chinese Academy of Sciences, Beijing, China; 3 College of Applied Sciences, Beijing University of Technology, Beijing, China; Beijing Normal University, Beijing, China

## Abstract

Neuroimaging community usually employs spatial smoothing to denoise magnetic resonance imaging (MRI) data, e.g., Gaussian smoothing kernels. Such an isotropic diffusion (ISD) based smoothing is widely adopted for denoising purpose due to its easy implementation and efficient computation. Beyond these advantages, Gaussian smoothing kernels tend to blur the edges, curvature and texture of images. Researchers have proposed anisotropic diffusion (ASD) and non-local diffusion (NLD) kernels. We recently demonstrated the effect of these new filtering paradigms on preprocessing real degraded MRI images from three individual subjects. Here, to further systematically investigate the effects at a group level, we collected both structural and functional MRI data from 23 participants. We first evaluated the three smoothing strategies' impact on brain extraction, segmentation and registration. Finally, we investigated how they affect subsequent mapping of default network based on resting-state functional MRI (R-fMRI) data. Our findings suggest that NLD-based spatial smoothing maybe more effective and reliable at improving the quality of both MRI data preprocessing and default network mapping. We thus recommend NLD may become a promising method of smoothing structural MRI images of R-fMRI pipeline.

## Introduction

Partial Differential Equation (PDE), a well-established mathematical theory, has given its advances on denoising images in terms of the strong theoretical framework with simple and efficient numerical strategies [1]. There are three main PDE-derived filters used to denoise or smooth images: Gaussian smoothing (isotropic diffusion equation, ISD) [2], anisotropic diffusion equations (ASD) [3] and non-local means diffusion (NLD) [4] (see [5], [6] for reviews). As a consequence of isotropic diffusion, the ISD is optimal in flat parts of the image but edges and texture are blurred. The ASD attempts to avoid the drawback of ISD by smoothing the image at a pixel only in the direction orthogonal to its gradient (i.e., smoothing along with edges). Both ISD and ASD are local smoothers and hard to preserve some global features of images (e.g., texture or periodic pattern). To address this issue, the NLD smoothes an image by taking into account the similarity of the geometrical configuration in a whole neighborhood (i.e., a patch of the image).

Currently, the ISD (i.e., a Gaussian smoothing kernel or heat kernel) is the most popular method used to reduce noise in structural and functional images of both 3D brain volume [7] and 2D cortical surface [8], [9]. The effect of applying the ASD to structural MRI data analysis have also been examined in both brain volume [10] and surface [11], [12]. Most recently, researchers have started to employ the NLD to denoise 3D structural brain images and presented its performance [13]–[16]. Although very rarely, the NLD was also applied to restore cortical surfaces based on their level sets [17]. While discrepancies of the smoothing performance between volume- and surface-based structural brain image analysis were widely investigated [18], [19], the direct comparison between the three spatial smoothing technics seems missing. As an initial effort along this direction, using structural MRI images from three subjects, we recently demonstrated the advantages of NLD in brain extraction, segmentation and registration for volume-based MRI analysis [20]. However, to our best knowledge, there is no statistical comparison on the performance of the three PDE-based smoothing of structural MRI data and the impact on the subsequent functional MRI analysis at a group level.

Here, we collected MRI data from 23 normal healthy controls and performed such comparisons by using volume-based brain image analysis (of note, same comparisons can be done on the cortical surface). Specifically, we first applied ISD, ASD and NLD to smooth individual T1 brain images to statistically evaluate their abilities to remove image noise and subsequent effects of structural brain processing. Three common steps of processing structural MRI data were chosen to demonstrate smoothing effects: brain extraction [21], tissue segmentation [22], and registration [23]. Second, according to the fact that these three structural processing steps are normally served as common steps of preprocessing resting-state functional MRI (R-fMRI) data [24], we thus mapped out the posterior cingulate cortex (PCC)-seeded default networks by using each of three smoothing filters in the structural preprocessing of an R-fMRI analysis pipeline and performed statistical comparisons between each other to evaluate the impact of different structural smoothing effects on R-fMRI analyses. Finally, to assess if these smoothing methods can improve the long-term test-retest reliability of PCC-anchored default network, two repeated R-fMRI measures were collected for each of nine participant separated by one year (i.e., one-year test-retest).

## Materials and Methods

### 1. Participants and imaging procedure

Twenty-three participants were scanned on a Phillips Achieva 1.5 Tesla scanner. For each participant, a high-resolution T1 anatomical image was obtained (TR = 7.1 ms; TE = 3.2 ms; 160 slices; FOV = 256 mm) and 240 EPI (TR = 2.0 s; TE = 50 ms; thickness/gap = 5 mm/1 mm; 22 slices; FOV = 230 mm; total scan time = 8 min6 s) R-fMRI images were collected. Among these subjects, nine subjects were scanned twice separated by one year, i.e., a one-year test-retest design. Of note, to keep the scanner's settings as consistent as possible between the test/retest scans, no any updates of hardware/software occurred to the scanner during the one-year duration. All participants are college students from Henan University of Traditional Chinese Medicine and had no history of psychiatric or neurological illness, as confirmed by clinical assessments. All subjects gave written, informed consent to participate in the study, which was approved by the Neuroimaging Acupuncture Research Center of Henan University of Traditional Chinese Medicine.

### 2. PDE-based spatial smoothing theory

The intensity of an MRI image can be defined in a bounded domain 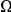 of 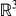 and denoted by 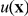 for 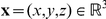. We use 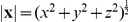 and 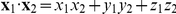 as the norm and scalar product. 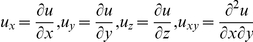 are the derivates of 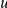. The gradient of 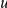 is written as 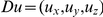 and the Laplacian of 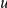 as 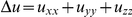.

The ISD (i.e., Gaussian smoothing) of image 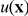 is characterized as in the equation below,
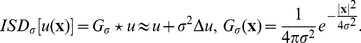
(1)In equation (1), 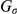 is a Gaussian kernel with standard deviation 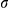 and 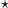 denotes a convolution operation. The smoothing operation is mathematically equivalent to solve the classic heat PDE 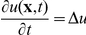, leading to weighted sum at each voxel.

A gaussian smoothing is optimal for harmonic functions. However, it performs poorly on edges or texture where its Laplacian is large. To avoid this drawback (i.e., blurring effect), ASD smoothes the image 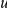 at 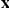 only in the direction orthogonal to 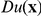:
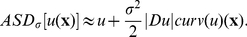
(2)In equation (2), 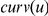 denotes the curvature. Similarly, the image smoothed with the ASD is a solution of the curve motion PDE 
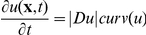
.

Both ISD and ASD are local neighborhood filters, which mean they average the intensity of voxels within a small spatial neighborhood. With such a strategy, it is difficult to maintain image texture – not a local feature of images. NLD addresses this problem by generalizing the diffusion domain to whole image domain 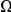. The similarity between two voxels 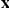 and 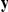 will be based on the similarity of the intensity gray level between their neighbors and computed with a Gaussian distance encoded as a kernel function
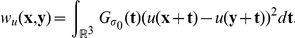
According to this distance, let 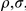 and 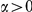 such that 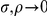 and 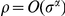. Define two functions as
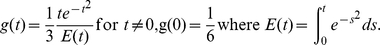
and
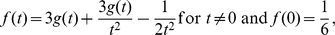
then NLD is
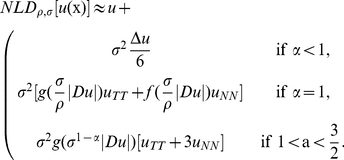
(3)where 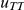 and 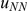 are tangent and orthogonal to the level line passing through 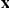, respectively. In theory, the NLD operation can be thought as the behavior of a non-local heat equation [25]:
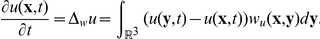
There seems no public neuroimaging package implementing all the three PDE-based smoothing methods. Accordingly, we carried out the above three spatial smoothing of individual T1 data from three publicly free software packages, respectively: 1) FMRIB Software Library (FSL: http://www.fmrib.ox.ac.uk/fsl, version 4.1) consisting of various comprehensive tools for brain imaging data, 2) Analysis of Functional NeuroImaging (AFNI: http://afni.nimh.nih.gov/afni, version 2011_05_26_1457) mainly designed for fMRI analysis and 3) Voxel-based morphometry (VBM) extent of Statistical Parametric Mapping (SPM: http://dbm.neuro.uni-jena.de/vbm/download, version 8). Another reason of using FSL and AFNI is that they both are the packages employed in the R-fMRI pipeline of our interest [24]. Specifically, the ISD was performed with a parameter 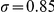 mm which corresponds to a 2 mm FWHM Gaussian kernel smoothing using the command fslmaths in FSL. It numerically implements ISD as Gaussian kernel weighted mean filtering. The ASD used a parameter 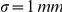 which is implemented in AFNI by using the command 3danisosmooth with 2 iterations and other default settings. This command employs a scheme for coherence-enhancing diffusion filtering with optimized rotation invariance based on an additive operator splitting (AOS) numerical strategy that leads to simple linear systems of equations [3], [26]. The NLD was performed by using a command cg_sanlm from the VBM8 toolbox [27]. It implemented an adaptive version of the optimized block-wise NLD to deal with spatially varying noise [15]. The optimized block-wise NLD uses particle swarm optimization based on partial least squares modeling to extend classical NLD on 2D images to that on 3D images by automatically tuning the smoothing parameter, selecting the most relevant voxels, dividing brain space into blocks and parallelizing computation to reduce the complexity of computation [13].

### 3. Structural MRI analysis: brain extraction, registration, segmentation

To evaluate the effect of three spatial smoothing approaches, we chose the three most frequently used preprocessing steps in both anatomical and functional MRI applications and quantify their performance on the denoised data: 1) brain extraction (BET) is widely employed as a preprocessing step in both computational anatomy and functional MRI analyses [24], [28]; 2) spatial normalization or registration is a process of matching an individual anatomical brain to a standard brain, which is a key part of group-level statistical analyses requiring all individual data to be in the same anatomical space; 3) brain tissue segmentation classifies the brain into three different tissues including grey matter (GM), white matter (WM) and cerebrospinal fluid (CSF). Such segmentation is a key to provide tissue references for functional MRI studies. Of note, BET as the first step is widely used as an initial processing in brain registration and segmentation. However, here, we extracted the brain manually to provide a golden standard of the brain extraction. This golden brain mask can extract brains from T1 images smoothed by ISD, ASD and NLD for subsequent brain registration and segmentation to exclude the impact of brain extraction quality on the two processes.

The brain extraction was done by using a command bet in FMRIB Software Library (FSL) [7] with parameters of ‘-f 0.3 -m –R’. Specifically, for each spatial smoothing method, the denoised T1 data were used as the inputs of bet. The original T1 data were also betted to show the impact of noise on the brain extraction (i.e., raw smoothing). To evaluate the effects of spatial smoothing, we proposed a quantitative index
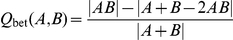
where 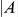 means the number of nonzero values in a brain mask 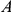 which includes 0 or 1 values. Obviously, 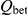 is 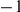 if there is no overlap between brain masks 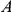 and 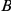 as well as 1 if 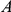 and 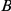 are perfectly overlapped. Of note, 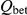 is similar to the common index for measuring the overlap rate, such as dice coefficient 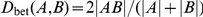. In fact, there is a monotonically increasing relationship between the two indices:
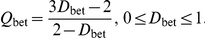
Given the golden brain mask 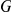 and a brain mask 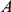 based on any of three spatial smoothing methods, 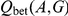 evaluates the effect of denoise on brain extraction performance.

To check the effects of these denoising methods on spatial normalization, for each smoothing approach, a fully automated robust and accurate tool for linear registration (12-parameter affine and spatial correlation-based cost-function) between individual T1 brains and the standard MNI152 brain was first computed in FMRIB's Linear Image Registration Tool (FLIRT). Based on this affine transformation, FMRIB's Non-linear Image Registration Tool (FNIRT) spatially normalized the T1 brain to match the standard brain by using a local spline basis deformation model [23]. It is difficult to find an objective index measuring the brain registration quality. Accordingly, for each T1 brain data from three participants, we compute the spatial correlation 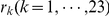 between normalized individual T1 brain 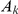 and the MNI152 standard brain 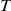;
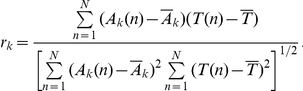
In above euqation, 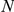 is the number of voxels in the MNI152 standard brain 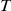 and 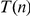 represents its intensity at the 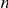-th voxel. We visually inspected the registration quality in terms of the important brain gyri and sulci.

FMRIB's Automated Segmentation Tool (FAST) was used to segment the T1 image into three tissues [22] with parameters of ‘-t 1 -g –p’. The FAST command based on a hidden Markov random field model and an associated Expectation-Maximization algorithm is fully automated and can also produce a bias field-corrected input image and a probabilistic and/or partial volume tissue segmentation. Again, the original T1 brain data were also fed into FAST to show the noise effect on the brain tissue segmentation. For each of 23 participants (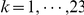), the distributions of each type tissue partial volume estimation (PVE) 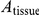 were calculated for evaluating spatial smoothing effects
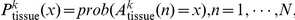
where tissue can be GM, WM or CSF, 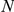 is the number of voxels in the T1 brain. Specifically, to explore how different smoothing strategies change PVE values of brain tissues, we estimated the histogram of partial volume estimation (PVE) by using five bins (i.e., 0–0.2; 0.2–0.4; 0.4–0.6; 0.6–0.8; 0.8–1.0).

For each of the three structural processing (i.e., brain extraction, registration and segmentation), paired t-tests were performed to show if there is any statistical difference in above measures between each pair of smoothing approaches.

### 4. Functional MRI analysis: mapping default network

The 1000 Functional Connectomes Project scripts http://www.nitrc.org/projects/fcon_1000 was selected as our R-fMRI pipeline of mapping resting state functional networks [24]. To evaluate the overall impact of PDE-based smoothing filters on the R-fMRI pipeline, we chose the well-known default network as our target of comparisons [29]. For each participant, image preprocessing comprises both anatomical and functional processing steps. Specifically, the anatomical processing steps included: 1) removal of non-brain tissue based on the anatomical images using the brain extraction tool in FSL, 3) automated segmentation of the GM, WM and CSF based on extracted brains, 4) a two-step registration of the high-resolution anatomical image to the MNI152 standard brain space: first, a 12-degrees-of-freedom linear affine transformation from individual brain image to the template was computed using FLIRT. Subsequently, combining the head images, the registration was refined using FNIRT nonlinear registration.

Functional preprocessing includes: 1) discarding the first 5 EPI volumes from each scan to allow for signal equilibration, 2) slice timing correction, 3) 3D motion correction, 4) co-registration between individual functional and anatomical brain images using a 6-degrees-of-freedom linear affine transformation, 5) spatial smoothing (6 mm FWHM Gaussian kernel), 6) 4D mean-based intensity normalization, 7) band-pass temporal filtering (0.01–0.1 Hz), 8) removal of linear and quadratic trends, 9) removal of nine nuisance covariates (signals from WM, CSF, the full brain, and six motion parameters). The resultant 4D residual time series was used for subsequent mapping of participant-level default network. The final maps of individual default network were spatially normalized to the 3 mm MNI152 standard space.

For each smoothing method, we first employed it to denoise all individual T1 images. The denoised T1 images were then fed into the R-fMRI pipeline of producing individual default network maps. The core seed of default network was adopted from [30] with the coordinates (−8,−56,26) in MNI standard brain space to map resting-state functional connectivity (RSFC) between each voxel and the seed. The group-level default network was generated by a one-sample t-tests on all individual default networks by using the R-fMRI pipeline with raw T1 images (i.e., no smoothing processing). We then performed three paired comparisons on individual default network maps across 23 subjects: ISD vs Raw, ASD vs Raw and NLD vs Raw. Whole-brain correction for multiple comparisons was performed (min Z
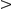
2.3; cluster significance: p
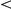
0.05, corrected).

### 5. Functional MRI analysis: test-retest reliability

Using one-year test-retest R-fMRI datasets from the 9 subjects, we assess if any of the three spatial smoothing methods can improve the test-retest reliability of default network mapping [31]. As in our prior work [32]–[36], we computed intra-class correlation coefficients (ICC) to quantify test-retest reliability. To calculate the ICC for each voxel, we consider a random sample of 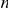 subjects with 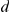 repeated measurements of a continuous variable 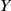 characterizing the default network RSFC with PCC. We denote 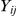 as the 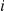-th measurement made on the 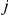-th subject (for 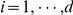 and 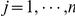). In the current situation, 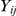 denotes the default network RSFC from the 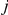-th participant's 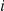-th measuring occasions. We apply a two-level linear mixed model to each voxel as the following decomposition of 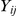:
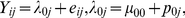
(4)where 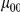 is a fixed parameter and 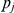 and 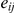 are independent random effects normally distributed with mean 0 and variances 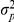 and 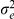. The term 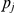 is the participant effect and 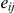 is the measurement error. The ICC was defined as
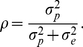
(5)Obviously, the ICC has the desired property to characterize the test-retest reliability, i.e., becoming smaller when 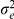 become larger. To avoid negative and get more accurate estimation of sample ICC, the variance components in above linear mixed-effects model (4) were estimated with the restricted maximum likelihood (ReML) approach built in SAS PROC MIXED [37]. The above voxel-wise ICC computing procedure was implemented in scripts combining MATLAB (Mathworks, Natick MA: reading and writing 3D brain volumes) and SAS (SAS Institute Inc., Cary NC: fitting mixed-effects linear model) commands.

## Results

**Figure 1:**
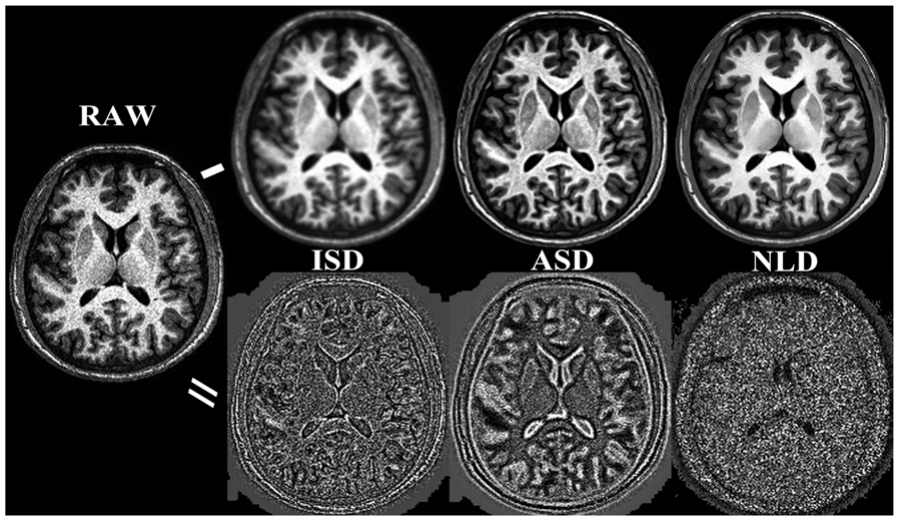
The effect of PDE-derived smoothers on an Individual Degraded Structural Image. PDE-based MRI denoising. An individual T1 structural image (RAW) were denoised by three PDE-based smoothing kernels: ISD, ASD and NLD. The first row shows the denoised T1 brain images as well as the second row depicts the difference images between each of the three smoothers and the raw noisy image.

As we demonstrated in our previous work [20], applied to real degraded raw T1 image (RAW), the NLD outperforms both ISD and ASD smoothers (Figure 1). Specifically, the ISD largely reduces the sharpness of GM-WM tissue boundaries while suppressing the noise, depicted as the overall boundary pattern in the difference image. By contrast, the ASD does good job in preserving the boundaries. However, it still distorts textures which is clearly presented in the ASD-RAW difference images. Moreover, the ASD can also introduce flow-like artifacts along tissue boundaries. As expected, the NLD produces rather uniform noise distributions, achieving promising removal of noise.

### 1. Effects of denoising: structural data processing

**Figure 2:**
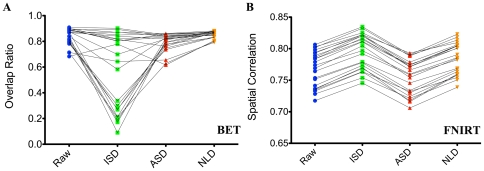
PDE-based Smoothing on Brain Extraction and Registration. For each of smoothing methods (Raw, ISD, ASD, NLD), scatter plots of overlap ratio and spatial correlation for all 23 participants were depicted.

To demonstrate the impact of different smoothing methods on brain extraction of T1 images, we extracted the brain masks from each smoothed T1 image and calculated overlap ratios 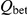 between the image and the mask via manual extraction. Scatter plots in Figure 2A depicted the overlap values for all 23 participants. Paired two-sample t-tests revealed that NLD and ASD exhibited significantly higher overlap ratio than ISD (p
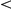
0.05). Clearly, ISD increases the individual variability of brain extraction. In contrast, NLD produces the smallest individual variability of overlap ratio though it did not show significant extraction improvements than raw data.

Brain registration via FNIRT (a locally non-linear process) resulted in different matching quality when the various methods of spatial smoothing were applied to denoise T1 brain data (Figure 2B). ASD demonstrated the worst performance (i.e., the significantly lower spatial correlation). Both ISD and NLD showed significantly higher spatial correlation than raw data (p
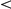
0.05; Figure 2B). This could be related to various artifactual edge-like flows introduced into the smoothed image by ASD.

**Figure 3:**
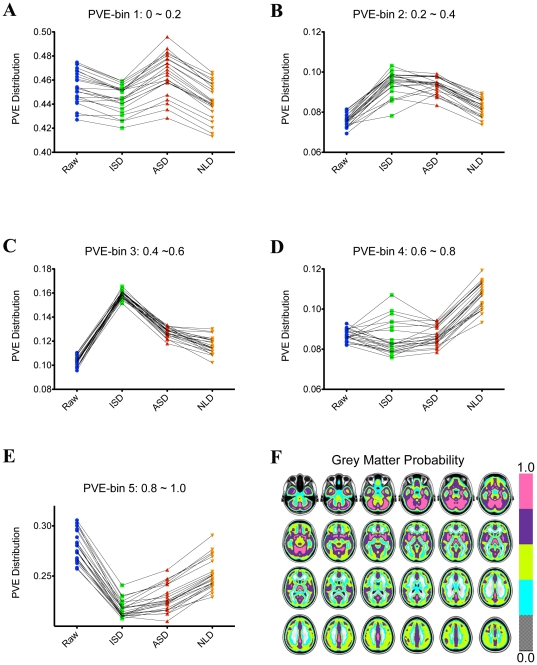
PDE-based Smoothing on Brain Tissue Segmentation. Scatter plots of probability of partial volume estimation for grey matter of 23 subjects. The histogram of partial volume estimation (PVE) using 5 bins (i.e., 0–0.2; 0.2–0.4; 0.4–0.6; 0.6–0.8; 0.8–1.0) indicate five different brain tissues (F): white matter and CSF (A), grey-white matter boundary (B), level-1 grey matter (C), level-2 grey matter (D) and level-3 grey matter (E).

As indicated by the grey matter probability map in Figure 3F, above five bins correspond to different brain tissues: white matter and CSF, grey-white matter boundary, level-1 grey matter, level-2 grey matter and level-3 grey matter. Our statistical analyses showed that NLD most significantly increases the PVE amount within level-2 grey matter tissues (Figure 3D) while both ISD and ASD significantly increase PVE distribution within grey-white matter boundary (Figure 3B) and level-1 grey-matter (Figure 3C). This is not difficult to understand because of the local nature of ISD and ASD smoothing filters, which is contrast to global feature of NLD smoothing. Of note, NLD have been demonstrated very effective to improve PVE recently [38].

### 2. Effects of PDE denoising: mapping default network

**Figure 4:**
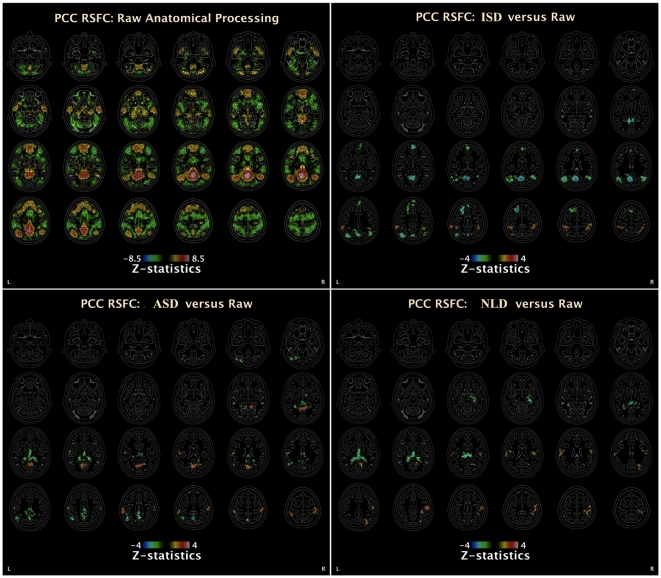
PDE-based Smoothing on Default Network Mapping. The group-level default network was generated by a one-sample t-tests on all individual default networks by using the R-fMRI pipeline with raw T1 images (i.e., no smoothing processing). We then performed three paired comparisons on individual default network maps across 16 subjects: ISD vs Raw, ASD vs Raw and NLD vs Raw. Whole-brain correction for multiple comparisons was performed (min Z
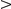
2.3; cluster significance: p
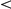
0.05, corrected).

Consistent with many previous R-fMRI studies (see [29] for a review), our R-fMRI pipeline with raw T1 data generated the default network presenting both positive and negative functional connectivity with PCC seed (Figure 4A). Figure 4B showed significant changes of RSFC introduced by performing ISD on T1 images, decreasing functional connectivity with four key default network areas: posterior cingulate cortex/precuneus (PCC/PCU), medial prefrontal cortex (MPFC) and bilateral inferior parietal cortex (IPC). In contrast, ASD reduced both artifactual (mainly subcortical regions around ventricles) and default network (PCC/PCU) RSFC as well as increases of RSFC with a little part of ventral PCC. Finally, NLD enhanced default network connectivity in PCC without reducing of the default network connectivity. However, as an advantage, it clearly suppressed the artifactual connectivity.

### 3. Effects of PDE denoising: test-retest reliability

**Figure 5:**
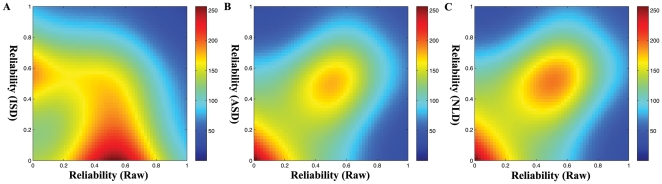
PDE-based Smoothing on Test-Retest Reliability. Two-dimensional histogram of ICC values. Each ICC map of the three PDE-based smoothing approaches was plotted versus the Raw ICC map: (A) ISD versus Raw, (B) ASD versus Raw, and (C) NLD versus Raw.

Using ReML-based ICC, the test-retest reliability maps of default network connectivity maps generated by the R-fMRI pipeline with the three spatial smoothing were calculated. Figures S1, S2, S3, S4 depicted all reliable voxels (i.e., ICC
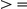
0.5) for default network with Raw, ISD, ASD and NLD smoothing respectively. To explore the differences in test-retest reliability among smoothing strategies, we first constructed a mask to include all reliable voxels for any of four smoothing ways. That is, the voxel showing ICC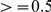 in any of four final ICC maps will be included in the mask. Second, all pairs of ICC within the mask between Raw and each of ISD/ASD/NLD smoothing were used to generate a 2-dimensional histogram [39]. As in Figure 5A, ISD indicated the most inconsistent spatial distribution of ICC values with the Raw ICC map, displaying as the non-diagonal hot coloring pattern. ISD also led to reduced test-retest reliability. In contrast, both ASD and NLD generated consistent spatial distribution of ICC with the Raw ICC map. But, different from ASD, NLD produced more extent and higher ICC values (Figure 5B and Figure 5C).

## Discussion

We statistically compared the differences in performance of three PDE-based spatial smoothing for MRI image processing and demonstrated the feasibility of the non-local mean diffusion technique in denoising T1 images and in improving accuracy and test-retest reliability of MRI image processing. The NLD approach tends to not only increase robustness of structural MRI processing (e.g., brain extraction, segmentation and registration) but improve the quality and reliability of mapping default network. It is particularly valuable to adopt NLD in fMRI studies because these studies often use T1 data polluted by unknown scanning noise from a low-strength magnetic field (e.g., 1.5T). Indeed, as we present in our previous study [20] and Figure 1 here, such denoise procedure could produce high quality T1 images similar to that from 3.0T scanner for cortical surface reconstruction [28].

Although NLD has been extensively studied in several 2D image processing fields, only a few recent studies have demonstrated the utility of NLD for effectively denoising MRI datasets. In [40], NLD exhibited the potential to reduce the Rician noise in diffusion-weighted MRI (DW-MRI). Similarly, Liu et al. also demonstrated the advantages of enhanced NLD to reduce Rician noise in MRI images [16]. Automatic NLD-based MRI denoising was systematically investigated in [14], illustrating significant benefits over other denoising methods. Nevertheless, despite its ability to remove noise, NLD is limited in computational speed due to its global search process in three-dimensional brain space. An optimized block-wise NLD was proposed in [13] to overcome this drawback. More recently, this optimized NLD was extended to deal with spatially-varying noise in MRI images [15].

A limitation of the current study is that we did not compare the performance of PDE-based filters by directly applying them to R-fMRI data. To our best knowledge, there is no study to examine the impacts of directly applying NLD to fMRI images. In contrast, there was a study employing ASD for detection of fMRI activation [41]. According the fact that fMRI images are temporal signals, it must be very interesting, particularly for NLD, to see how these spatial smoothing filters change patterns of spin labeling or T2* weighted BOLD R-fMRI activity, which will be further explored in our future work.

In summary, our study confirmed the utility of non-local diffusion equations in denoising degraded T1 MRI images. This approach demonstrates a promising potential to improve various fundamental MRI analytic processes including brain extraction, tissue segmentation, registration and subsequent functional MRI analyses. NLD method could serve as an initial preprocessing step in future MRI studies.

## Supporting Information

Figure S1
**One-year Test-Retest Reliability Maps for Default Network Mapping with Raw Structural Smoothing.** This figure depicts the voxel-wise one-year test-retest reliability of PCC-derived resting-state functional connectivity or default network. The axial views of the reliability maps are displayed in radiological convention. The ICC map is thresholded at ICC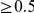, with a minimum cluster size of 20 voxels.(TIF)

Figure S2
**One-year Test-Retest Reliability Maps for Default Network Mapping with ISD Structural Smoothing.** This figure depicts the voxel-wise one-year test-retest reliability of PCC-derived resting-state functional connectivity or default network. The axial views of the reliability maps are displayed in radiological convention. The ICC map is thresholded at ICC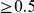, with a minimum cluster size of 20 voxels.(TIF)

Figure S3
**One-year Test-Retest Reliability Maps for Default Network Mapping with ASD Structural Smoothing.** This figure depicts the voxel-wise one-year test-retest reliability of PCC-derived resting-state functional connectivity or default network. The axial views of the reliability maps are displayed in radiological convention. The ICC map is thresholded at ICC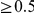, with a minimum cluster size of 20 voxels.(TIF)

Figure S4
**One-year Test-Retest Reliability Maps for Default Network Mapping with NLD Structural Smoothing.** This figure depicts the voxel-wise one-year test-retest reliability of PCC-derived resting-state functional connectivity or default network. The axial views of the reliability maps are displayed in radiological convention. The ICC map is thresholded at ICC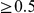, with a minimum cluster size of 20 voxels.(TIF)
