# Correction: Effects of Non-Local Diffusion on Structural MRI Preprocessing and Default Network Mapping: Statistical Comparisons with Isotropic/Anisotropic Diffusion

**DOI:** 10.1371/annotation/dd83fe6c-b41e-4c8a-8430-7f2e6ea6b5de

**Published:** 2011-11-10

**Authors:** Xi-Nian Zuo, Xiu-Xia Xing

The correct funding information is: "The current study was partially supported by the Startup Foundation for Distinguished Research Professor of Institute for Psychology (Y0CX492S03), the National Nature Science Foundation of China (10671131, 81171409), Youth Foundation of Beijing University of Technology (X1006012201001) and Natural Science Foundation for Basic Research of Beijing University of Technology (JX006012201002). The funders had no role in study design, data collection and analysis, decision to publish, or preparation of the manuscript." 

