# Supplementary figures and images for: Effects of Non-Local Diffusion on Structural MRI Preprocessing and Default Network Mapping: Statistical Comparisons with Isotropic/Anisotropic Diffusion
Source: PLoS One. 2011 Oct 31;6(10):e26703. doi: 10.1371/journal.pone.0026703 (PMC3204989; doi:10.1371/journal.pone.0026703)

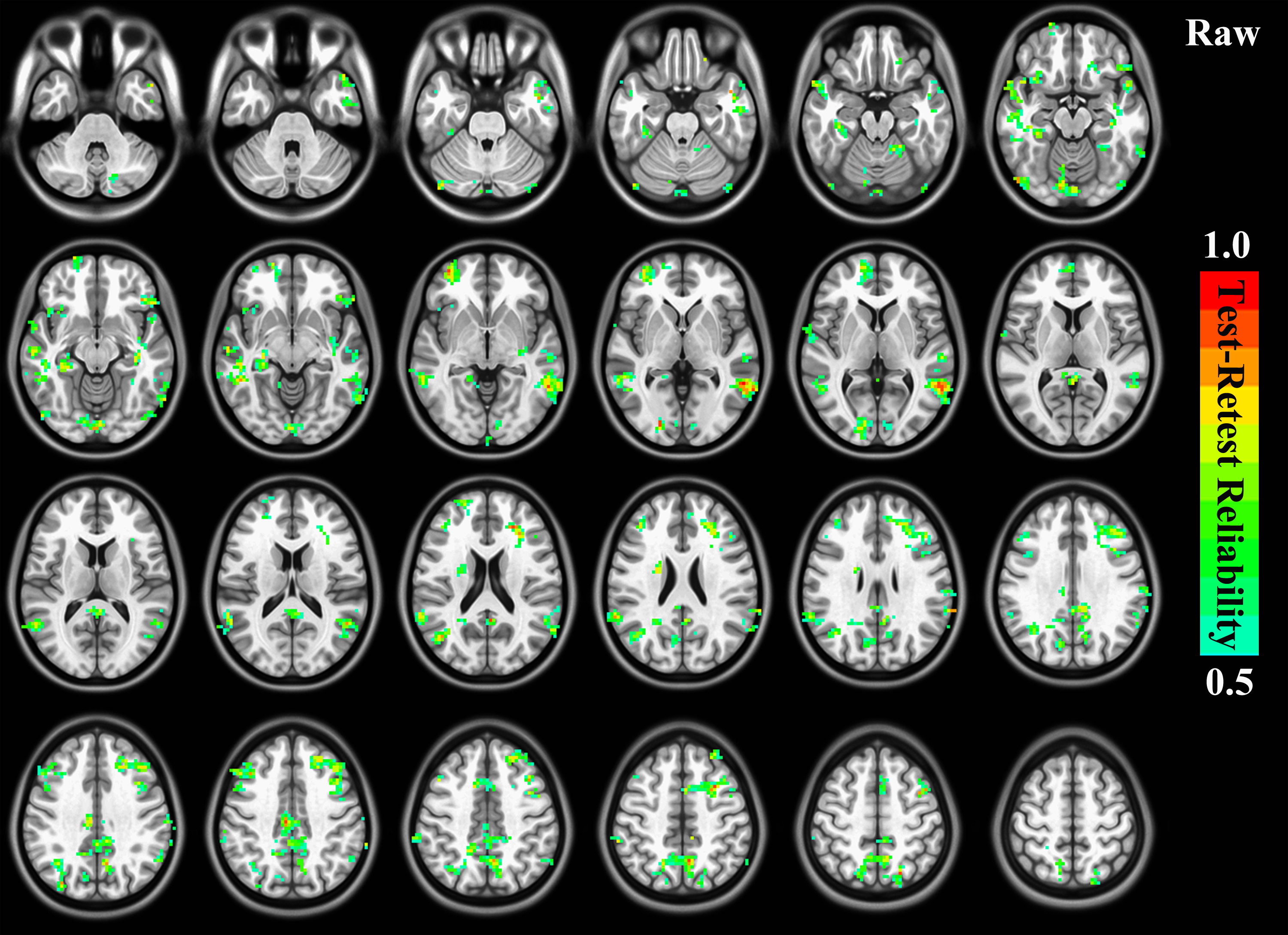

Supplement: Figure S1 — One-year Test-Retest Reliability Maps for Default Network Mapping with Raw Structural Smoothing. This figure depicts the voxel-wise one-year test-retest reliability of PCC-derived resting-state functional connectivity or default network. The axial views of the reliability maps are displayed in radiological convention. The ICC map is thresholded at ICC, with a minimum cluster size of 20 voxels. (TIF) [file pone.0026703.s001.tif]

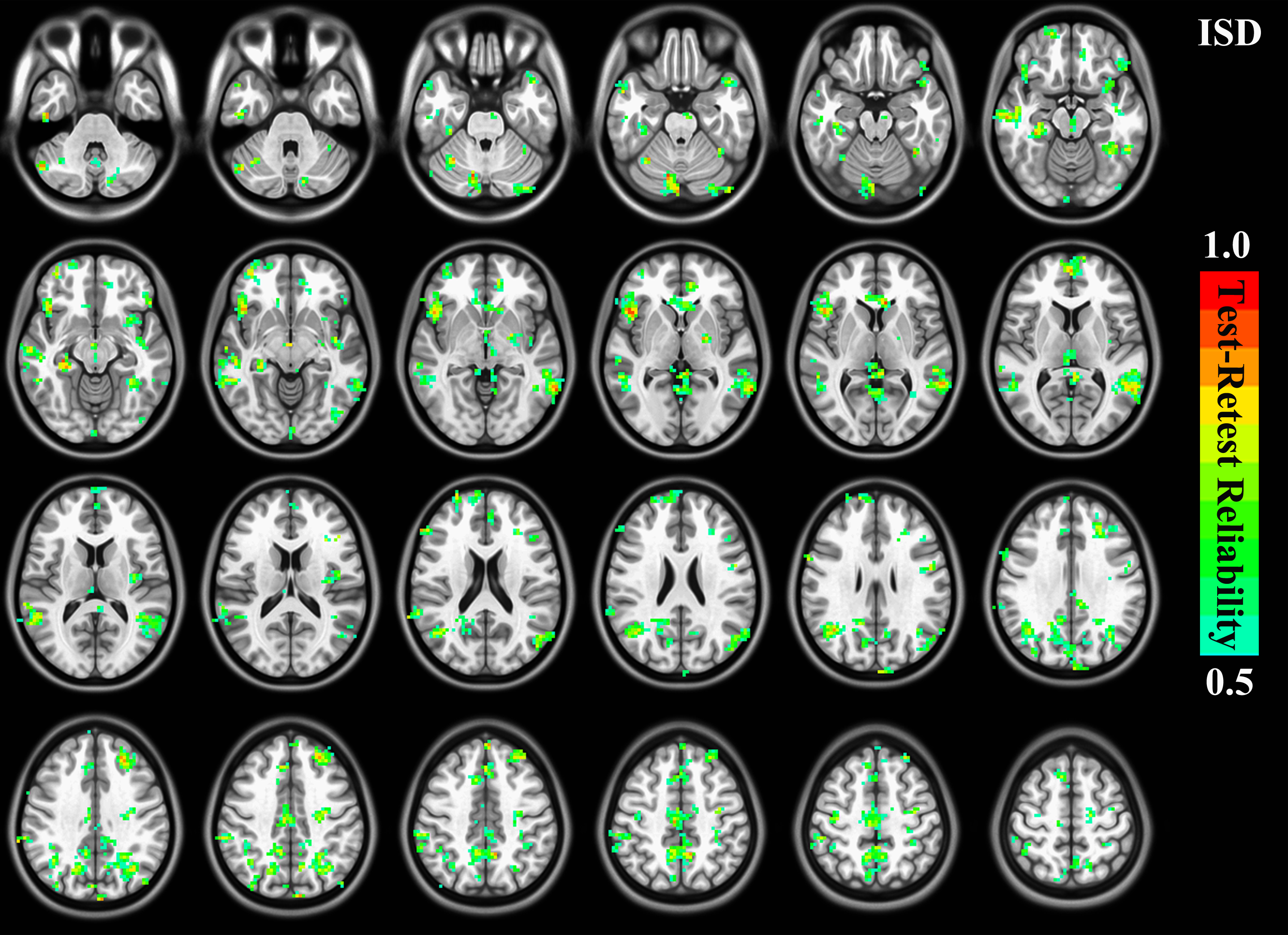

Supplement: Figure S2 — One-year Test-Retest Reliability Maps for Default Network Mapping with ISD Structural Smoothing. This figure depicts the voxel-wise one-year test-retest reliability of PCC-derived resting-state functional connectivity or default network. The axial views of the reliability maps are displayed in radiological convention. The ICC map is thresholded at ICC, with a minimum cluster size of 20 voxels. (TIF) [file pone.0026703.s002.tif]

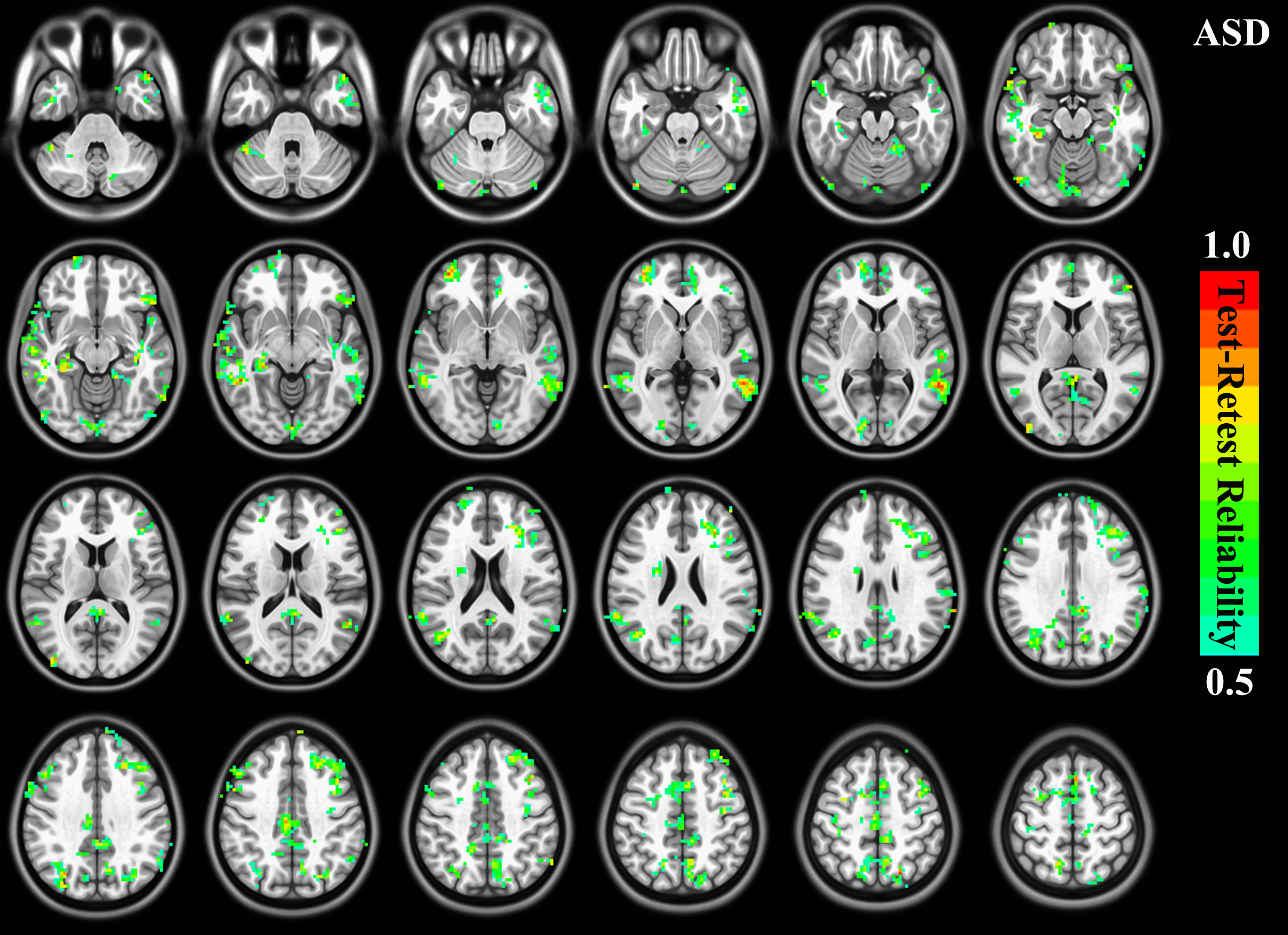

Supplement: Figure S3 — One-year Test-Retest Reliability Maps for Default Network Mapping with ASD Structural Smoothing. This figure depicts the voxel-wise one-year test-retest reliability of PCC-derived resting-state functional connectivity or default network. The axial views of the reliability maps are displayed in radiological convention. The ICC map is thresholded at ICC, with a minimum cluster size of 20 voxels. (TIF) [file pone.0026703.s003.tif]

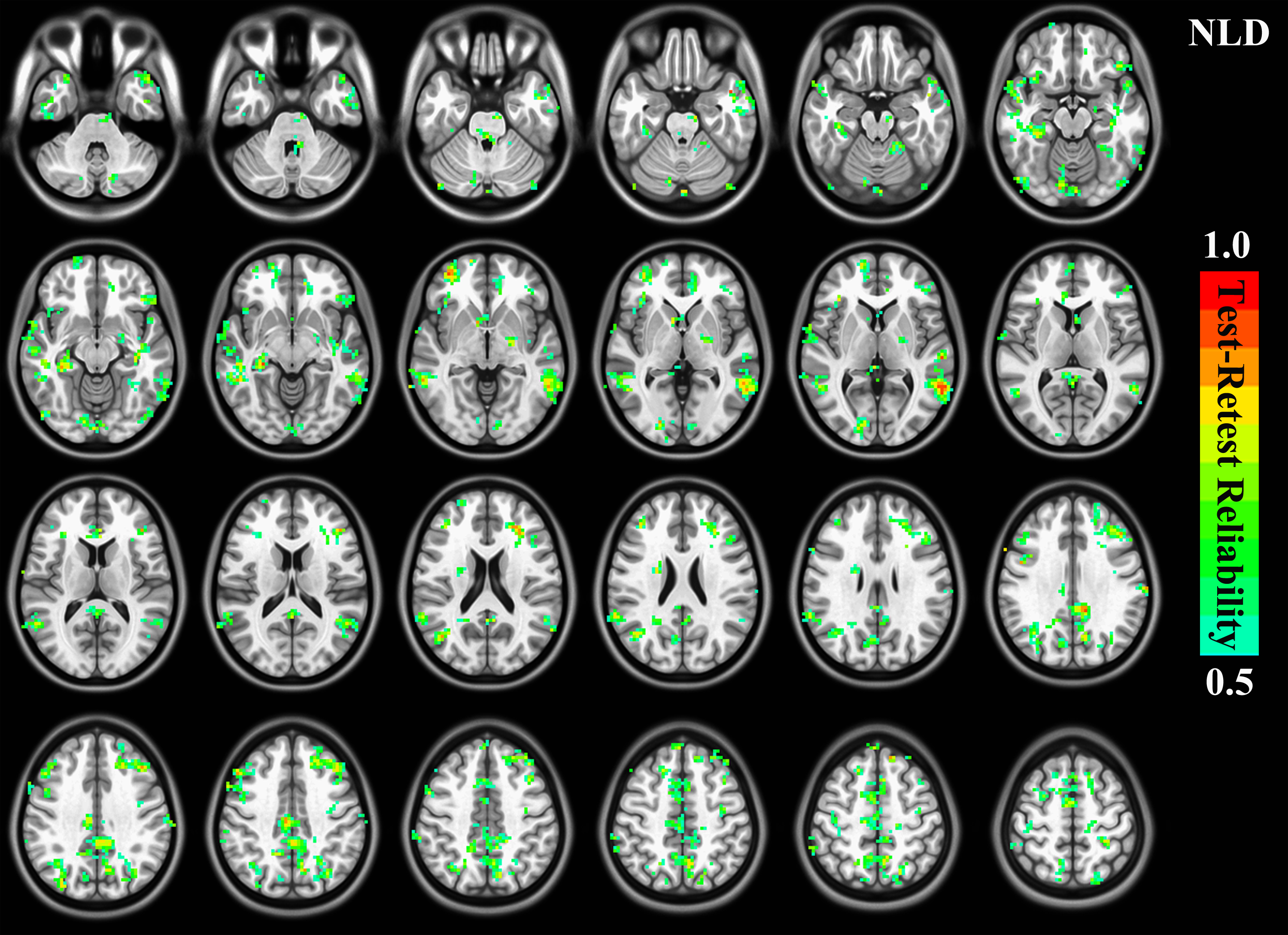

Supplement: Figure S4 — One-year Test-Retest Reliability Maps for Default Network Mapping with NLD Structural Smoothing. This figure depicts the voxel-wise one-year test-retest reliability of PCC-derived resting-state functional connectivity or default network. The axial views of the reliability maps are displayed in radiological convention. The ICC map is thresholded at ICC, with a minimum cluster size of 20 voxels. (TIF) [file pone.0026703.s004.tif]
